# Involvement of p38-βTrCP-Tristetraprolin-TNFα axis in radiation pneumonitis

**DOI:** 10.18632/oncotarget.17770

**Published:** 2017-05-10

**Authors:** Pranathi Meda Krishnamurthy, Shirish Shukla, Paramita Ray, Rohit Mehra, Mukesh K Nyati, Theodore S Lawrence, Dipankar Ray

**Affiliations:** ^1^ Department of Radiation Oncology, University of Michigan, Ann Arbor, MI, USA; ^2^ Department of Pathology, University of Michigan, Ann Arbor, MI, USA; ^3^ Current address: RNA Therapeutics Institute, University of Massachusetts Medical School, Worcester, MA, USA

**Keywords:** radiation pneumonitis, TNF-α, Tristetraprolin, p38 MAPK, β-TrCP1

## Abstract

Early release of tumor necrosis factor-alpha (TNF-α) during radiotherapy of thoracic cancers plays an important role in radiation pneumonitis, whose inhibition may provide lung radioprotection. We previously reported radiation inactivates Tristetraprolin (TTP), a negative regulator of TNF-α synthesis, which correlated with increased TNF-α release. However, the molecular events involved in radiation-induced TTP inactivation remain unclear. To determine if eliminating *Ttp* in mice resulted in a phenotypic response to radiation, *Ttp*-null mice lungs were exposed to a single dose of 15 Gy, and TNF-α release and lung inflammation were analyzed at different time points post-irradiation. *Ttp^−/−^* mice with elevated (9.5±0.6 fold) basal TNF-α showed further increase (12.2±0.9 fold, *p*<0.02) in TNF-α release and acute lung inflammation within a week post-irradiation. Further studies using mouse lung macrophage (MH-S), human lung fibroblast (MRC-5), and exogenous human TTP overexpressing U2OS and HEK293 cells upon irradiation (a single dose of 4 Gy) promoted p38-mediated TTP phosphorylation at the serine 186 position, which primed it to be recognized by an ubiquitin ligase (E3), beta transducing repeat containing protein (β-TrCP), to promote polyubiquitination-mediated proteasomal degradation. Consequently, a serine 186 to alanine (SA) mutant of TTP was resistant to radiation-induced degradation. Similarly, either a p38 kinase inhibitor (SB203580), or siRNA-mediated β-TrCP knockdown, or overexpression of dominant negative Cullin1 mutants protected TTP from radiation-induced degradation. Consequently, SB203580 pretreatment blocked radiation-induced TNF-α release and radioprotected macrophages. Together, these data establish the involvement of the p38-βTrCP-TTP-TNFα signaling axis in radiation-induced lung inflammation and identified p38 inhibition as a possible lung radioprotection strategy.

## INTRODUCTION

The efficacy of radiation therapy for upper thoracic cancers is limited by radiation pneumonitis. Amifostine, a free-radical scavenger and the only FDA-approved radioprotector, does not protect lung and carries its own substantial toxicity [1–3]. Thus, there is a need to develop a lung radioprotector. Various pro-inflammatory cytokines including IL-1β, IL-6, and TGF-β1 have been implicated in radiation-induced lung toxicity [4–6]. Besides, early release of tumor necrosis factor-alpha (TNF-α) is a critical factor [7, 8], and we have shown that blocking TNF-α signaling either via knockdown or using antisense oligonucleotides against the TNF receptor can protect mouse lung from radiation injury [9]. As long-term delivery of siRNA or antisense oligonucleotides remains a challenge, identification of novel targets that can be accessed using a small molecule would be important to limit RILT.

We previously identified an RNA binding anti-inflammatory protein, Tristetraprolin (TTP) as a major negative regulator of radiation-induced TNF-α synthesis by mouse lung macrophages [10]. We found radiation promotes TTP inactivation via phosphorylation and proteasomal degradation, which correlates with increased TNF-α synthesis and release. Thus, radiation therapy uses a relatively similar mechanism as LPS to cause TTP inactivation leading to enhanced TNF-α production [11, 12]. Initial studies utilizing different genetically modified mouse models have identified p38α-MK2 pathway as a major modulator of various inflammatory responses and cytokine production including LPS and TNF-α mediated inflammation [13, 14]. Similarly, such signaling has been directly linked to various inflammatory diseases including rheumatoid arthritis, multiple sclerosis, atherosclerosis and chronic obstructive pulmonary disease (COPD) [15–18]. Thus, p38 inhibitors are being tested in several clinical trials for anti-inflammatory efficacy. However, the involvement of p38 kinase activity in radiation-induced lung inflammation remains unclear, as does the impact of a p38 kinase inhibitor as a radioprotector.

Given the potential relationship between p38 kinase and TTP, we decided to obtain a better understanding of the physiological relevance of TTP inactivation in developing radiation pneumonitis and further identify molecular regulators of radiation-induced TTP inactivation and degradation. Specific phosphorylation of TTP at serine 186 by p38 MAPK inactivates its mRNA-destabilizing ability [19]. We also found that radiation-induces activation of p38 leading to phosphorylation of TTP at Ser^186^ [10]. While analyzing the amino acid sequence, we further noted that upon phosphorylation of Ser^186^, surrounding residues (^186^S^P^FSGLPS^192^) can potentially serve as a recognition motif (phospho-degron) for an F-box family ubiquitin ligase (E3), SCF^β-TrCP^(consensus D^p^SGxx^p^S, where x could be any amino acid) [20, 21]. As β-TrCP is known to be activated by radiation, which can induce degradation of certain proteins [22], we, therefore, wished to test the hypothesis that p38 MAP kinase inhibition could provide lung radioprotection via blocking radiation-induced Ttp phosphorylation and ubiquitination-dependent protein degradation.

In the present study, we used *Ttp* knockout mice to establish the physiological relevance of *Ttp* in radiation-induced lung inflammation. Furthermore, we provide evidence of a linkage between radiation-induced p38-mediated TTP phosphorylation and SCF^β-TrCP^-mediated polyubiquitination and degradation, which in coordination promote TTP inactivation allowing a TNF-α mediated lung inflammatory response. Using a pan p38 inhibitor, we then found inhibition of p38 to be radioprotective.

## RESULTS

### Ttp knockout mice are susceptible to radiation-induced pneumonitis

**Figure 1:**
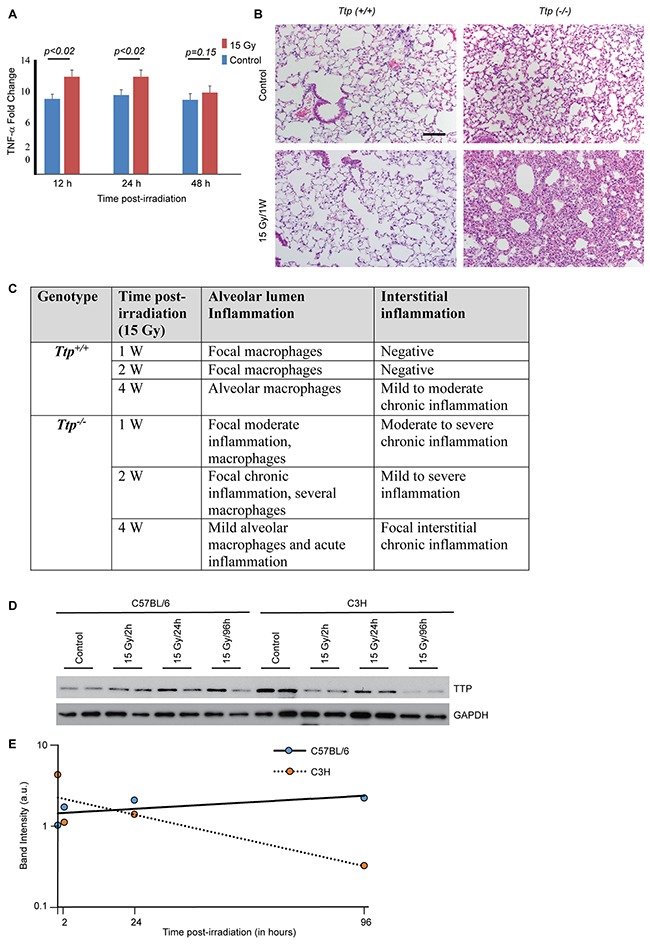
*Ttp* knockout mice are susceptible to radiation-induced lung inflammation **(A)** Fold change in TNF-α in BAL either from sham-irradiated (control) or 15 Gy single exposure of *Ttp^−/−^* mice compared to *Ttp^+/+^* after indicated times post-irradiation (n=6 mice for each time point for each genotype category). **(B)** The thoraxes of *Ttp*^+/+^ or *Ttp^−/−^* mice were either sham-irradiated (- RT) or exposed to 15 Gy. Shown is representative H&E staining of paraffin-embedded lung sections. Scale bar, 200 μm. **(C)** Summary of histopathological observation in *Ttp^+/+^* and *Ttp^−/−^* mice upon 15 Gy whole thorax irradiation (n=5 mice for each time point for each genotype category). **(D)** C57BL/6 and C3H mice strains were either sham-irradiated or 15 Gy single exposure of upper thorax. Lung specimens were collected after 2, 24 and 96 hours post-irradiation and cryo-preserved. Following completion of the study, cryosections were subjected to protein isolation as described in the materials and methods and subjected to immunoblotting for total Ttp protein. GAPDH was used as loading control. **(E)** Average band intensity (arbitrary units) was calculated from two representative samples from each group (as shown in panel D) using Image J software and plotted against time post-irradiation.

We began our studies by irradiating both the lungs of either *Ttp^+/+^* or *Ttp^−/−^* mice (C57BL/6 background) with a single 15 Gy dose. These mice were observed for a month (n=6 per group) after irradiation. While analyzing TNF-α levels we noted the basal level of TNF-α to be higher (9.54±0.67 fold, p<0.0001) in bronchoalveolar lavage (BAL) of *Ttp^−/−^* as compared to *Ttp^+/+^* mice (Figure 1A), which is consistent with a previous report [23]. Consequently, histopathological examination of sham-irradiated *Ttp^−/−^* mice lungs showed multi-focal chronic inflammation at the alveolar lumens and interstitial spaces with occasional focal septal thickening compared to sham-irradiated *Ttp^+/+^* mice. An increased number of macrophages was also noted in the alveolar lumen in the knockout lung (Figure 1B, upper panel). Further, upon 15 Gy single dose irradiation, a measurable increase (12.2±0.85 fold, p<0.02) in TNF-α levels were noted in *Ttp^−/−^* mice (n=6 per group) within 12-24 hours post-irradiation, which started decreasing by 48 hours (10.2±0.85 fold, p=0.15) (Figure 1A). In irradiated *Ttp^−/−^* mice, acute lung tissue damage became evident within a week showing focal acute and chronic inflammation in the alveolar lumen with very severe and acute chronic inflammation in the interstitial spaces (Figure 1B, lower panel). In contrast, at the same time point, the irradiated wild-type mice showed a minimal increase in focal macrophages in the alveolar lumen with marginal changes in interstitial inflammation (Figure 1B, lower panel). Detailed pathological analyses revealed normal alveolar epithelium, vessels and pleura in all animals and there was no sign of bronchiolar epithelium damage. Lung specimens collected and analyzed for histopathology at 1, 2, and 4 weeks post-irradiation (n=6/time point) showed persistent inflammation in *Ttp^−/−^* mice during this observation period, as summarized in Figure 1C. From these data, we conclude that *Ttp* is a negative regulator of radiation-induced lung inflammation in mice.

Mouse strains are known to respond differently to ionizing radiation; C3H mice are prone to radiation-induced inflammation, whereas, C57BL/6 mice are fibrosis prone [24, 25]. The *Ttp*^−/−^ mice used in the study were in a C57BL/6 background and despite their C57BL/6 background showed acute inflammation in response to radiation thus raising the possibility of Ttp as an important determinant. To examine whether ionizing radiation has any differential impacts on Ttp levels in different strains of mice, C57BL/6 and C3H mice upper thoraxes were irradiated with a single dose of 15 Gy. As shown in Figure 1D and quantified in Figure 1E, a faster decay of Ttp proteins were noted in C3H mice within 4 days of irradiation.

### SCF^β-TrCP^ is the ubiquitin ligase involved in radiation-induced TTP degradation

**Figure 2:**
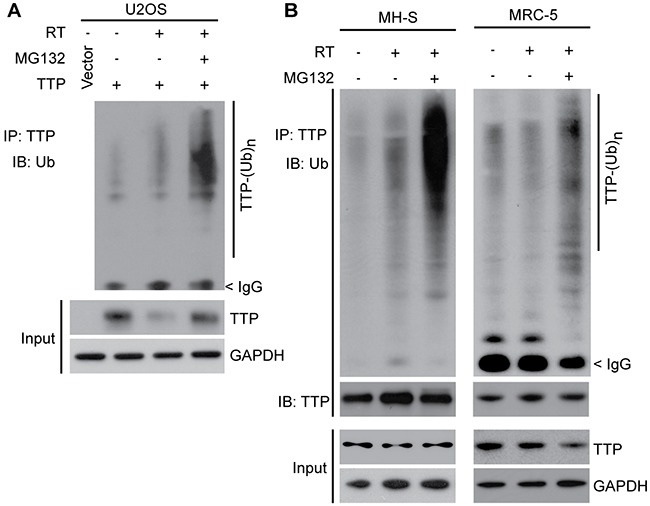
Ionizing radiation induces polyubiquitination-mediated proteasomal degradation of TTP **(A)** U2OS cells overexpressing human TTP were sham irradiated or exposed to 4 Gy and were either left untreated for the next 24 h or treated with 2 μM MG132 for the last 4 h. Cell lysates were subjected to immunoprecipitation using TTP antibody followed by immunoblotting using ubiquitin (Ub) antibody. **(B)** MH-S (left panel) and MRC-5 (right panel) cells were irradiated as above. Cells were then either left untreated or 6 h after irradiation treated with 2 μM MG132 for the next 4 hours. Cell lysates were then subjected to immunoprecipitation and immunoblotting using indicated antibodies.

As *Ttp* knockout mice were found to be sensitive to radiation pneumonitis, and as we previously reported that radiation causes TTP protein inactivation primarily via enhanced inhibitory phosphorylation as well as via inducing protein degradation [10], we next focused our studies on improving the mechanistic understanding of TTP degradation machinery. To ascertain whether radiation-induced TTP protein degradation is mediated via ubiquitination-mediated proteasomal degradation, we utilized both overexpression and endogenous systems. We previously reported that in a mouse lung macrophage cell line (MH-S), radiation-induced *Ttp* protein down-regulation can be blocked using a proteasome inhibitor MG132 [10]. Here we have obtained similar data using U2OS cells overexpressing human TTP protein (Figure 2A). Furthermore, a concomitant increase in TTP polyubiquitination was noted in the presence of MG132 in both the systems when TTP was immunoprecipitated using a specific antibody (Figure 2B, left panel). Similar data were obtained in normal human lung fibroblasts (MRC-5) (Figure 2B, right panel). Together, these data show that radiation induces polyubiquitination-mediated proteasomal degradation of TTP.

**Figure 3:**
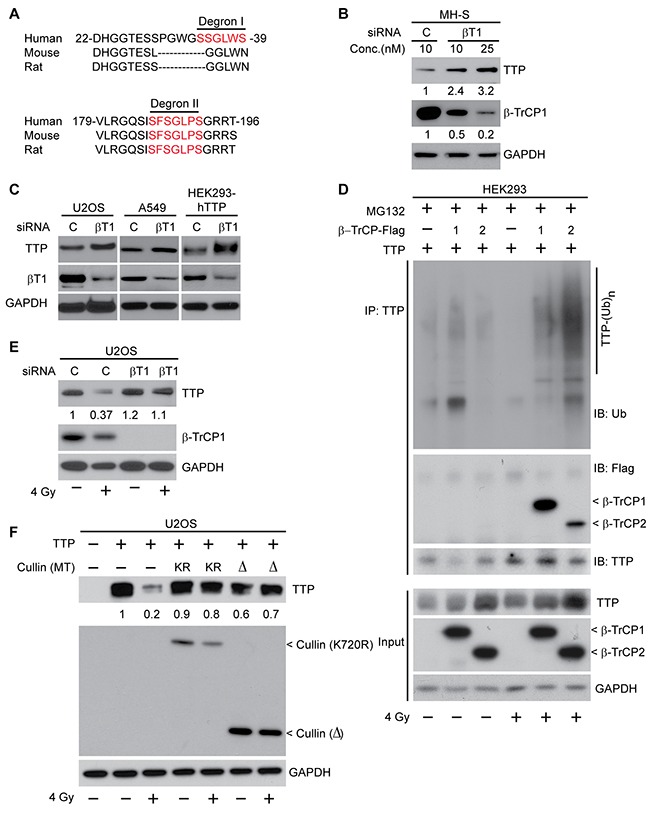
SCF^β-TrCP^ is the ubiquitin ligase involved in radiation-induced TTP degradation **(A)** Amino acid sequence alignment between human, mouse and rat TTP showing the presence of two putative β-TrCP recognition motifs (degron I and degron II). **(B)** MH-S cells were transfected either with a control or with two different concentrations (10 and 25 nM) of β-TrCP1 siRNA. Forty eight hours post-transfection cell lysates were subjected to immunoblotting. Fold changes in TTP and β-TrCP1 were calculated based on band intensity using Image J software. **(C)** U2OS and A549 cells with endogenous TTP or HEK293 cells overexpressing human TTP were transfected either with control or β-TrCP1 siRNA. Cell lysates were prepared 48 h post-transfection and immunoblotted. **(D)** HEK293 cells overexpressing human TTP in the presence or absence of either β-TrCP1 or β-TrCP2 overexpression as indicated were either sham irradiated or irradiated with 4 Gy. Six hours later cells were treated with MG132 as above. Cell lysates were then immunoprecipitated using TTP antibody and immunoblotted. **(E)** U2OS cells were transfected either with control or β-TrCP1 siRNA and 24 h post-transfection were either sham-irradiated or irradiated with 4 Gy. Twenty four hours post-irradiation, cell lysates were immunoblotted. Changes in total TTP levels were calculated assuming control siRNA treated sample band intensity as 1. **(F)** U2OS cells overexpressing human TTP were co-transfected with various Cullin-1 mutants [either K720R or a Δ (1-327) mutant] as indicated. Cells were irradiated as above, and 24 h post-irradiation, cell lysates were immunoblotted. Changes in total TTP levels were calculated assuming TTP overexpressed untreated control band intensity as 1.

To identify the ubiquitin ligase (E3) responsible for radiation-induced TTP ubiquitination, we looked for the presence of a degron (degradation signal) present in TTP protein, which can be recognized by an E3 enzyme. Among various E3s, an F-box family ligase, beta transducing repeat containing protein (β-TrCP), is known to recognize a phospho-degron consisting of the DS^p^GxxS^p^ motif, where ‘x’ could be any amino acid, and serine (S), when phosphorylated (P), becomes a recognition motif [20, 21]. Additionally, β-TrCP family E3 ligases are known to be activated by radiation to degrade proteins including CDC25A [22]. While analyzing the TTP primary amino acid sequence we identified two probable phospho degrons (Figure 3A). ‘Degron I’ spans the N-terminal part between amino acids 34-39, and the ‘degron II’ spans between amino acids 186-193. To ascertain the role of β-TrCP in radiation-induced TTP ubiquitination, we used β-TrCP1 specific siRNA and transfected MH-S cells with different concentrations of siRNA. As shown in Figure 3B, a dose-dependent decrease in β-TrCP1 levels resulted in a corresponding increase in TTP protein. We found similar responses in other cell lines including human osteosarcoma U2OS, human lung adenocarcinoma A549 and even in human embryonic kidney HEK293 cells overexpressing human *TTP* cDNA (Figure 3C). To further understand the involvements of β-TrCP1 and its homolog β-TrCP2, we utilized HEK293 cells transiently overexpressing human TTP in the presence or absence of either β-TrCP1 or β-TrCP2. In sham-irradiated cells, TTP immunoprecipitation followed by immunoblotting using either ubiquitin (Ub) or DDK (tag used to detect both b-TrCP1 and 2) antibodies showed minimal changes in TTP ubiquitination and no detectable interactions between TTP and β-TrCP1/2-DDK (Figure 3D, lanes 1-3). However, there was a significant increase in TTP interaction with β-TrCP1/2, which resulted in the increase of TTP polyubiquitination within 6 hours of 4 Gy exposure (Figure 3D, lanes 4-6). Similarly, a pooled siRNA-mediated knockdown of β-TrCP protected radiation-induced TTP downregulation (Figure 3E). As Cullin plays an important role in β-TrCP mediated substrate polyubiquitination and degradation, we tested two different Cullin-1 mutants (K720R and 1-327 deletion), which are known to inhibit β-TrCP activity [26]. In Figure 3F, we demonstrate that radiation-induced TTP downregulation can be inhibited by overexpressing either of the two Cullin-1 mutants. We, therefore, conclude that upon irradiation, TTP is recognized by β-TrCP via phospho-degron(s) leading to ubiquitination-mediated proteasomal degradation.

**Figure 4:**
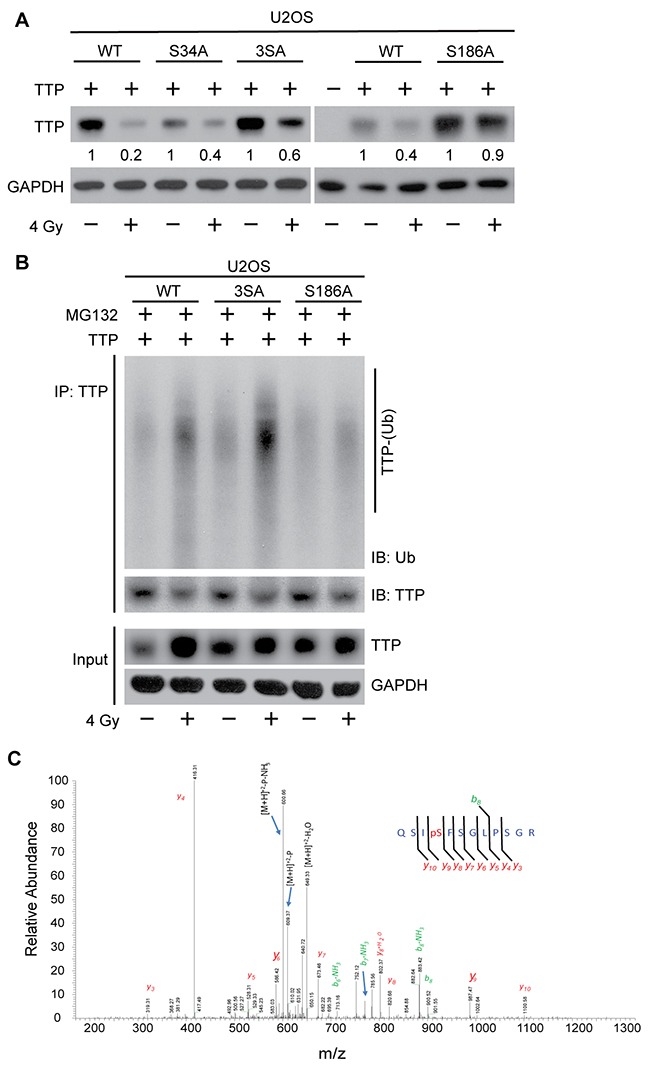
Radiation-induced Ser^186^ phosphorylation of TTP primes it for polyubiquitination **(A)** U2OS cells overexpressing either wild-type (WT) or various serine to alanine mutants [S34A, S34/35/39A (3SA) and S186A] of human TTP, were either sham irradiated or treated with 4 Gy. Twenty-four hours post-irradiation cell lysates were immunoblotted. Changes in total TTP levels were calculated assuming TTP overexpressed untreated control band intensity as 1. **(B)** U2OS cells overexpressing either WT or various (3SA and S186A) mutants of TTP were either sham-irradiated or treated with 4 Gy. Six hours after irradiation cells were treated with MG132 as above, and 4 h post-MG132 treatment cell lysates were immunoprecipitated using TTP antibody and immunoblotted. **(C)** Representative MS/MS spectrum identifying serine^186^ phosphorylation on TTP upon 4 Gy.

To further understand the importance of the two probable degrons in radiation-induced TTP degradation, we carried out site-directed mutagenesis at both degrons (I and II). As shown in Figure 4A (left panel), serine (S) to alanine (A) substitution (SA) at degron I had a minimal effect on radiation-induced TTP downregulation. In contrast, a mutation of S186A provided significant protection of radiation-induced decay (Figure 4A, right panel). Similarly, the TTP (S186A) mutant showed minimal change in radiation-induced polyubiquitination (Figure 4B, compare lanes 2, 4 and 6). Thus, degron II of TTP appears to be primarily responsible for substrate recognition by β-TrCP for radiation-induced polyubiquitination and degradation. In our previous study [10] (Figure 2E) we reported that overexpression of S186A mutant TTP can block radiation-induced TNF-α release by irradiated lung macrophages (MH-S), suggesting the functional significance of this phosphorylation.

### Radiation-induced p38-mediated Ser^186^ phosphorylation of TTP primes it for degradation

Previous studies have reported that p38-mediated serine 186 phosphorylation promotes functional inactivation of TTP, which is essential for increased stabilization of various cytokine transcripts including TNF-α [12, 27, 28]. As we showed previously that radiation induces p38-mediated TTP phosphorylation at S186 both *in vitro* and *in vivo*, and as now we have found that S186A mutant TTP is resistant to radiation-induced ubiquitination and degradation, we hypothesized that, in the context of radiation the S186 phosphorylation and β-TrCP mediated ubiquitination and degradation are interconnected. To further establish radiation effects on S186 phosphorylation of TTP, we utilized a U2OS overexpression system expressing wild-type human TTP. Cells were exposed to 4 Gy, and cell lysates were immunoprecipitated using TTP antibody and subjected to mass spectrometry analyses. As shown in Figure 4C, we could detect an S186 phosphorylated fragment only in the irradiated sample, supporting the previous observation. Taken together, these data show that radiation induces Ser186 phosphorylation of TTP to prime it for β-TrCP recognition followed by polyubiquitination and proteasomal degradation.

### P38 inhibitor (SB203580) provides radioprotection of cytokine producing macrophages, but not for lung cancer cells

**Figure 5:**
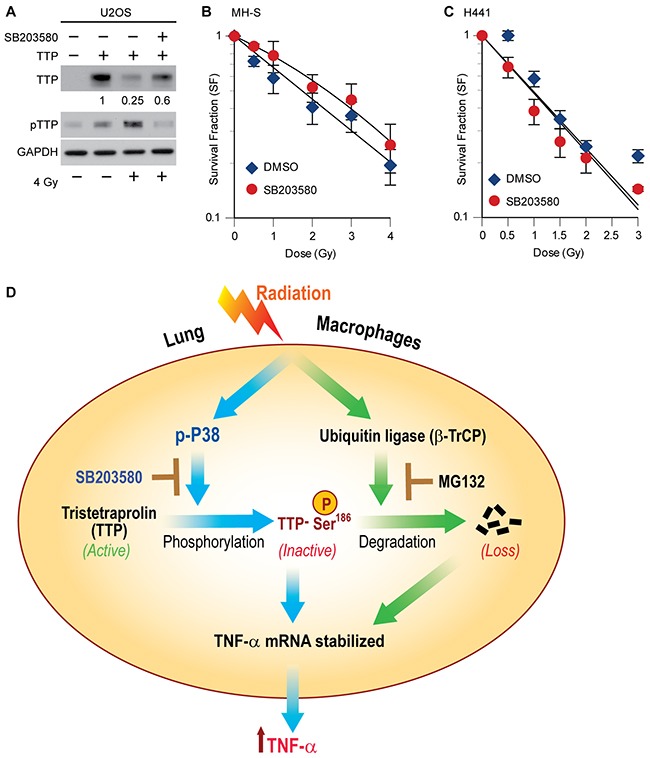
P38 inhibitor (SB203580) blocks radiation-induced TTP degradation and radioprotects lung macrophages **(A)** U2OS cells overexpressing human TTP were either treated with DMSO or 1 μM SB203580 thirty minutes prior to 4 Gy. Twenty-four hours after irradiation cell lysates were prepared and immunoblotted. Change in total TTP levels were calculated assuming TTP overexpressed untreated control band intensity as 1. **(B, C)** Either MH-S cells (in B) or H441 cells (in C) were plated at clonal density and after 24 h treated with 1 μM of SB203580 thirty minutes prior to different doses of radiation. Cells were then left for colony formation as described in materials and methods. **(D)** Schematic model showing the interconnectedness between p38-mediated phosphorylation and SCF^β-TrCP^-mediated degradation in radiation-induced TTP inactivation and further proposing the novel use of the p38 inhibitor as a lung radioprotector. Previously reported observations are shown using solid blue lines, whereas, data shown in the current manuscript are denoted using solid green lines.

Previously, we have shown SB203580 pretreatment can inhibit radiation-induced TTP phosphorylation and TNF-α secretion by MH-S cells [10]. Similarly, U2OS cells overexpressing human TTP when treated with the SB compound prior to radiation, also blocked TTP degradation (Figure 5A). As the addition of TNF-α is known to radiosensitize cells [29, 30], we, therefore, considered the possibility that a p38 inhibitor could radioprotect lung macrophages by blocking TNF-α release. To test our hypothesis, we pretreated mouse lung macrophage cells (MH-S) plated at clonal density with SB203580 thirty minutes prior to different doses of radiation and assessed colony formation ability. As shown in Figure 5B, an improved survival of macrophages was noted when they were irradiated in the presence of SB203580, with a radiation enhancement ratio (ER) of 0.86. Importantly, similar pretreatment of SB203580 using human lung adenocarcinoma cells (H441) did not provide any radioprotection (ER 1.03) (Figure 5C). Thus, we identified a novel radioprotective effect of SB203580 specific to cytokine producing lung macrophages with no effects on lung cancer cells.

## DISCUSSION

Previously, we have identified Tristetraprolin (TTP) as a negative regulator of radiation-induced TNF-α production by mouse lung macrophages [10]. Here, we report that upon genetic ablation of *Ttp*, mouse lungs were more susceptible to radiation-induced inflammation (pneumonitis), which is correlated with increased TNF-α levels. These findings suggest that Ttp may be a ‘negative’ regulator of radiation-induced lung inflammation in mice, primarily via controlling the early release of TNF-α. Previously [10], we have identified two primary ways ionizing irradiation can inactivate TTP: (i) via p38-mediated inhibitory phosphorylation and (ii) via proteasome-mediated degradation. Here, we have identified a linkage between the two processes; mass spectrometry showed that radiation causes TTP phosphorylation at serine^186^ (a known p38-mediated phosphorylation site), which is then recognized by SCF^β-TrCP^ E3 ligase to promote polyubiquitination and proteasomal degradation. Such concerted phosphorylation and degradation-mediated inactivation of TTP releases its anti-inflammatory effects, allowing the secretion of TNF-α by irradiated lung macrophages. A schematic model summarizing our findings is shown in Figure 5D.

As reported previously, *Ttp* knockout mice secrete higher levels of various pro-inflammatory cytokines, particularly TNF-α, resulting in inflammatory responses in tissues including arthritis, which can be mitigated by injecting anti-TNF-α neutralizing antibody [23]. In this manuscript, we report for the first time that *Ttp* knockout mice are also prone to radiation-induced lung inflammation. Radiation pneumonitis is reported to be mouse strain-dependent; C3H-HeN mice are more prone to radiation pneumonitis, whereas, C57BL/6 mice are not [24, 31]. Importantly, the *Ttp^−/−^* mice used in this study had a C57BL/6 background. After giving 15 Gy to the whole-lung, we noted acute lung inflammation within a week which was sustained for at least during the observation period of 4 weeks. In a recent study using C57BL/6 and C3H strains, it was demonstrated that radiation exposure can cause differential elevation of alveolar and interstitial macrophages at different time points, which may be responsible for differential outcomes [32]. However, no molecular insight has been provided for such differential macrophage activation. Our *in vivo* findings raised the possibility that Ttp plays an important role in blocking early phenotypes of radiation-induced lung pneumonitis in the C57BL/6 as compared to the C3H-HeN strain. Our data (Figure 1D, 1E) further support such a hypothesis, where we noted faster decay of Ttp protein particularly in C3H mice. However, we acknowledge that the use of a single strain, rather than multiple strains, is a limitation of our study.

The possibility of TTP's involvement as a predictor of radiation pneumonitis emphasized our rationale to identify molecular regulators controlling the levels of TTP proteins. TTP protein is inherently unstable and undergoes proteasomal degradation upon radiation [10]. However, regulators including the ubiquitination machinery involved in maintaining lower TTP protein steady state levels in cells remain unclear. Previous studies have shown that, following TNF-α treatment, Tumor Necrosis Factor Receptor-associated Factor 2 (TRAF-2), an ubiquitin ligase (E3), is responsible for K63-linked polyubiquitination of TTP [33]. However, such ‘hypermodification’ of TTP seems to work as a non-degrading signal and is important in balancing the activation of NF-κB and JNK signaling cascades to determine cell fate. In another study, a member of the Cullin family (Cullin-4B), a known component of the CRL4 ubiquitin ligase complex, was found to interact with TTP upon LPS stimulation. Such association was reported to be critical for the TTP-TNF-α mRNA ribonucleoprotein (mRNP) complex loading onto polysomes, which in turn prevents decay of TNF-α mRNA [34]. In fact, unlike radiation (which downregulates TTP), LPS treatment is known to upregulate TTP phosphorylation (functionally inactive) as well as protein steady state levels [35, 36] and may be mediated via the above mechanism. In comparison, our findings of radiation-induced TTP protein degradation is unique and the identification of β-TrCP as an ubiquitin ligase is novel. Interestingly, a previous study has shown that LPS treatment can mitigate radiation-induced lung injury in knockout mice of either TNF-α or its receptors in the C57BL/6J background [37]. Thus, one could speculate that LPS-induced compensatory upregulation of functionally inactive (phosphorylated) Ttp can still inhibit radiation-induced lung inflammation, although this needs to be tested.

Our study further expands the role of β-TrCP in inflammation and cytokine responses. For example, the role of β-TrCP in the degradation of IκBα leading to the activation of an inflammatory transcription factor, NF-κB [38] is well established. Upon LPS treatment, β-TrCP promotes inflammation via polyubiquitination and degradation of an anti-inflammatory protein, called lysophosphatidylcholine acyltransferase 1 (LPCAT1) [39]. Similarly, in IL-17-mediated inflammation, β-TrCP degrades Act1, an adaptor protein associated with IL-17 receptor desensitization [40]. During TNF-α induced inflammation, β-TrCP1 promotes such activity via degrading an anti-inflammatory protein, silencing mediator of retinoic acid and thyroid hormone receptor (SMRT) [41]. Recently, β-TrCP was also implicated in inflammatory cytokine production via polyubiquitination and degradation of AUF1 [42], another ARE-binding cytokine mRNA decay factor, similar to TTP. We have previously demonstrated the downregulation of the anti-inflammatory TTP protein when mouse lungs were irradiated with a single dose of 15 Gy [10]. Here, using various cell biology and biochemical analyses, we have established the involvement of β-TrCP in radiation-induced TTP degradation that in turn promotes increased TNF-α release and lung inflammation.

The part of our study that is most clinically relevant is the identification of p38 as the kinase responsible for radiation-induced phosphorylation of TTP at serine 186 leading to functional inactivation as well as facilitation of β-TrCP-mediated polyubiquitination, and degradation, which allows stabilization of the TNF-α transcript. Such findings led us to hypothesize that a p38 inhibitor might protect TTP from radiation-induced post-translational modifications including phosphorylation and ubiquitination, thus could be used as a lung radioprotector. Our proof-of-principle study with a pan-p38 inhibitor (SB203580) supports such a hypothesis. Unfortunately, SB203580 cannot be tested as a lung radioprotector due to poor pharmacokinetics properties.

In conclusion, by utilizing *Ttp^−/−^* mice we demonstrated the *in vivo* anti-inflammatory role of Ttp in radiation pneumonitis. Our data identify p38 as a regulator of radiation-induced TTP inactivation and provide *in vitro* evidence that a pharmacologically favorable p38 inhibitor may warrant testing as a lung radioprotector in future preclinical and clinical studies.

## MATERIALS AND METHODS

### Ethics statement

Here we confirm that all the animal studies conducted here were approved by the University Committee on Use and Care of Animals (UCUCA) of the University of Michigan (protocol # PRO00005911).

### Cells and reagents

The mouse alveolar macrophage cell line MH-S, and human lung fibroblast MRC-5 cells were obtained from ATCC and cultured in regular DMEM supplemented with 10% FBS (Sigma). For overexpression studies easy-to-transfect human lung osteosarcoma (U2OS) and human embryonic kidney (HEK293) cells were also obtained from ATCC and cultured in DMEM medium with 10% FBS. Human lung adenocarcinoma (NCI-H441 and A549) cells were cultured in RPMI-1640 medium supplemented with 10% FBS. Rabbit polyclonal TTP antibodies were purchased from Abcam Inc. (ab36558 and ab83579). Mouse monoclonal anti-DDK antibody was purchased from Origene (Rockville, MD), and antibodies against β-TrCP1 and GAPDH were purchased from Cell Signaling Technology (Danvers, MA). Control (Cat. No. D-001810) and β-TrCP1 (Cat. No. D-003463) siRNA were purchased from Thermo Fisher Scientific (Lafayette, CO). P38 kinase inhibitor SB203580 (Cat. V1161) was purchased from Promega Corp. (Madison, WI).

### Constructs

PCMV-hTTP (wild type) construct was purchased from Origene Inc. and was further modified to in-frame fused FLAG-tag. To generate serine-to-alanine mutants (SA) of TTP [S34A, S34/35/39A (3SA) and S186A] wild-type construct was subjected to site-directed mutagenesis using the QuikChange II site-directed mutagenesis kit (Agilent Technologies, Santa Clara, CA).

### siRNA transfection

Cells were transfected using Lipofectamine RNAimax (Invitrogen) with 20 nM of either a control siRNA duplex or a siRNA duplex targeting β-TrCP1 using the protocol as described previously [43]. Twenty-four hours after the second transfection, cells were either sham radiated or irradiated with 4 Gy (Philips RT250 orthovoltage unit that produces 250 kV X-rays, Kimtron Inc., Oxford, CT), and the cell lysates were harvested and subjected to immunoblotting.

### Clonogenic survival assay

Clonogenic cell survival assays were performed as described previously [44]. Briefly, cells (either MH-S or NCI-H441) were plated in triplicate at a pre-determined cell density. Cells were then incubated overnight under normal culture condition and pretreated with p38 inhibitor (SB203580) one hour prior to different doses of radiation. Cells were then left for six to nine days for colony formation and were fixed (methanol and acetic acid 7:1) and stained using crystal violet solution (0.5% w/v). Surviving fraction following each radiation dose was normalized to sham-irradiated control cells and cell survival enhancement ratio (ER) was calculated as the ratio of the mean inactivation dose in the control divided by the irradiated cells.

### Mouse lung irradiation, BAL and tissue collection

A breeding pair of *Ttp ^+/-^* mice was obtained from Dr. Perry Blackshear's laboratory and intercrossed and genotyped to obtain *Ttp^−/−^* mice. Both the wild-type and mutant mice were maintained in a C57BL/6 background. Male mice 6 to 8 weeks of age were either sham irradiated or irradiated with a single dose of 15 Gy to the whole lung as described previously [9]. Bronchoalveolar lavage (BAL) was collected using PBS containing 1% FBS and the recovered fluids were centrifuged (400×g for 5 min at 4°C) and supernatants were stored at -80°C for ELISA. For tissue collection, the right ventricle of the heart was perfused with PBS to clear blood from the lungs which were then fixed in 10% buffered formalin. Samples were collected at different time points post-irradiation and subjected to histopathological analyses for pneumonitis.

### Mouse TNF-α ELISA

TNF-α secreted into the culture medium was quantified using an ELISA kit (R&D systems) according to the manufacturer's instructions.

### Protein analyses

For immunoblotting, cell lysates were prepared as described previously [45]. In brief, culture dishes were placed on ice and washed once with ice-cold PBS. The required amounts of lysis buffer were added to each plate, and cells were harvested by scraping. Cells were lysed using a cup-type sonicator followed by clearing of debris by centrifugation at 4°C. Protein concentration was quantified using Bradford reagents according to manufacturer's instructions.

To isolate proteins from OCT-fixed frozen lung sections, protocols were followed as described previously [46] with modifications. Briefly, OCT was dissolved by washing sections using PBS, followed by addition of 2X laemmli buffer with 2-marcaptoethanol. Samples were then sonicated and boiled for 10 minutes.

### Mass spectrometry

Mass spectrometry analyses of TTP protein to identify residues phosphorylated upon irradiation were performed as described previously. In brief, U2OS cells overexpressing FLAG-tagged wild-type TTP protein were either sham-irradiated or exposed to 4 Gy. Thirty (30) min post-irradiation, cell lysates were prepared and subjected to immunoprecipitation. Immuno-adsorbed proteins were then separated on a polyacrylamide gel, and proteins were visualized with colloidal coomassie stain. In-gel digestion followed by identification of phosphorylation site mapping was carried out essentially as described previously [47]. Briefly, upon trypsin digestion, peptides were resolved on a nano-capillary reverse phase column and subjected to high-resolution, linear ion-trap mass spectrometer (LTQ Orbitrap XL, Thermo Fisher). The full MS scan was collected in Orbitrap (resolution 30,000@400 m/z), and data-dependent MS/MS spectra on the nine most intense ions from each full MS scan were acquired. Proteins and peptides were identified by searching the data against the SwissProt human protein database, appended with decoy (reverse) sequences, using the X!Tandem/Trans-Proteomic Pipeline (TPP) software suite. All proteins identified with a ProteinProphet probability of >0.9 (for <1%) were accepted. Spectral matches to phosphorylated peptides were manually verified.

### Statistical analysis

Data are expressed as mean ± SE. The unpaired Student's t-test was used to compare the differences between two groups, and a *P* value of <0.05 was considered as significant.
